# Mapping in the era of sequencing: high density genotyping and its application for mapping TYLCV resistance in *Solanum pimpinellifolium*

**DOI:** 10.1186/1471-2164-15-1152

**Published:** 2014-12-20

**Authors:** Marcela Víquez-Zamora, Myluska Caro, Richard Finkers, Yury Tikunov, Arnaud Bovy, Richard GF Visser, Yuling Bai, Sjaak van Heusden

**Affiliations:** Wageningen UR Plant Breeding, Wageningen University & Research Centre, P.O. Box 386, Wageningen, AJ 6700 the Netherlands; Centre for Biosystems Genomics, P.O. Box 98, Wageningen, AB 6700 the Netherlands; Graduate School Experimental Plant Sciences, Wageningen, 6708 PB the Netherlands

**Keywords:** SNPs, *S. pimpinellifolium*, *In silico*, TYLCV, Flavonoids, Hexose, Genotype by sequencing (GBS)

## Abstract

**Background:**

A RIL population between *Solanum lycopersicum* cv. Moneymaker and *S. pimpinellifolium* G1.1554 was genotyped with a custom made SNP array. Additionally, a subset of the lines was genotyped by sequencing (GBS).

**Results:**

A total of 1974 polymorphic SNPs were selected to develop a linkage map of 715 unique genetic loci. We generated plots for visualizing the recombination patterns of the population relating physical and genetic positions along the genome.

This linkage map was used to identify two QTLs for TYLCV resistance which contained favourable alleles derived from *S. pimpinellifolium*. Further GBS was used to saturate regions of interest, and the mapping resolution of the two QTLs was improved. The analysis showed highest significance on Chromosome 11 close to the region of 51.3 Mb (*qTy-p11*) and another on Chromosome 3 near 46.5 Mb (*qTy-p3*). Furthermore, we explored the population using untargeted metabolic profiling, and the most significant differences between susceptible and resistant plants were mainly associated with sucrose and flavonoid glycosides.

**Conclusions:**

The SNP information obtained from an array allowed a first QTL screening of our RIL population. With additional SNP data of a RILs subset, obtained through GBS, we were able to perform an *in silico* mapping improvement to further confirm regions associated with our trait of interest. With the combination of different ~ omics platforms we provide valuable insight into the genetics of *S. pimpinellifolium-*derived TYLCV resistance.

**Electronic supplementary material:**

The online version of this article (doi:10.1186/1471-2164-15-1152) contains supplementary material, which is available to authorized users.

## Background

*Solanum pimpinellifolium* is a source for introgression breeding in tomato *(S. lycopersicum)*. This species is one of the closest wild relatives of *S. lycopersicum*, and it is present in the pedigree lineage of some commercial cultivars such as the sequenced ‘Heinz 1706’ [1]. Linkage maps from crosses between *S. lycopersicum* and *S. pimpinellifolium* were generated by various researchers [2–8]. Their work represents a small piece of the successful use of genome-wide linkage analyses to map underlying genetic factors of traits between the two species.

Recombinant inbred lines (RILs) derived from inter-specific crosses consist of individuals with parental mosaics and are an efficient resource for mapping quantitative trait loci (QTL) [9]. Genotyping with molecular markers allows the visualization of recombination patterns which is crucial for the elucidation of loci associated with segregating traits [10, 11]. This has become more efficient due to the availability of vast numbers of markers such as single nucleotide polymorphisms (SNPs). In tomato, the availability of high throughput SNP arrays allows massive parallel whole-genome screening of genotypes [8, 12].

Nowadays, next generation sequencing technologies are offering new ways to increase genotyping throughput by several orders of magnitude [13]. Even more, it is possible to combine different genotyping platforms to increase the power of the analyses. Furthermore, due to published complete tomato genomes [1], next generation re-sequencing approaches can be applied in related germplasm [14]. Studies on evolutionary and domestication, as well as the genetic basis underlying important traits can be benefited from these genomic tools [15].

TYLCV is the causal agent of an aggressive tomato disease that can result in production losses up to one hundred percent, and its rapid spread worldwide is threatening the production of tomatoes. Development of TYLCV resistant tomato cultivars is an important strategy to avoid the damage caused by TYLCV. However, no TYLCV resistance has been identified in the cultivated tomato germplasm, except for the resistance allele of *ty-5* which is possibly originated from a mutation in the cultivated tomato [16]. Breeding for resistance to TYLCV has been focused on the introgression of tolerance or resistance genes from tomato wild relatives such as *S. pimpinellifolium, S. chilense*, *S. habrochaites* and *S. peruvianum* [17, 18]. Several *S. pimpinellifolium* accessions are known to confer resistance to the virus [19–24], but attempts to map the causal factor in this species were not very successful. Thus, *S. pimpinellifolium*-derived TYLCV resistance is currently not well-exploited in tomato breeding programs [25]. In our study we genotyped a RIL population between *S. lycopersicum* cv. Moneymaker and *S. pimpinellifolium* G1.1554 with a custom made SNP array [12], and a subset of 60 lines was also genotyped by sequencing using Illumina HiSeq 2000 (150 Tomato Genome ReSequencing project; http://www.tomatogenome.net). Furthermore, we explored the population with an untargeted metabolic profiling and compared resistant vs. susceptible lines in order to get more insights on compounds that might play a role in the resistance. Our study shows how we can combine different ~ omics approaches to identify genetic loci underlying resistance to Tomato Yellow Leaf Curl Virus (TYLCV) in *S. pimpinellifolium* using a RIL population.

## Methods

### Recombinant Inbred Lines (RILs)

From a cross between *S. lycopersicum* cv. Moneymaker and *S. pimpinellifolium* G1.1554 (CGN reference CGN 15528) a set of 100 RILs was generated through single seed descent (SSD) until the six^th^ generation [26]. These RILs, which have been used for many different experiments e.g. Khan *et al.* [27], were used in this study.

### DNA extraction

Genomic DNA from young leaflets was extracted using a CTAB based protocol [28, 29] adjusted for high throughput isolation. Two young leaflets were ground with a Retsch 300 mm shaker (Retsch BV, Ochten, The Netherlands) using 1 ml micronic tubes (Micronic BV, Lelystad, The Netherlands). DNA pellets were washed in 76% EtOH with 10 mM NH_4_Ac before re-suspending the DNA in TE buffer.

### Genome wide genotyping

Genome wide genotyping was done as described by Víquez-Zamora *et al.* [12]. In short, DNA samples were sent to ServiceXS ( http://www.servicexs.com/), Leiden, The Netherlands. A custom made Infinium HD Ultra Assay protocol [30] was used for hybridization onto a BeadChip. The Genotyping Module 1.9.4 of Illumina’s GenomeStudio® V2011.1 software package was used to analyse the genotyping results under default settings. All samples corresponding to the RIL population and the parents were selected for a separate analysis in which manual inspection and adjustment were performed in order to discard questionable SNPs for the population and to optimize call rates. All polymorphic SNPs for the RIL population were named after their position on the SL2.40 version ( http://solgenomics.net/) of the tomato genome sequence published online [1].

### Genotype by sequencing (GBS)

A subset of 60 lines was selected for resequencing (lines with extreme values for TYLCV resistance were included). Whole genomic DNA was isolated from each line (see above). Shallow sequencing of 500 bp inserts was carried out using Illumina HiSeq 2000 (100 bp paired end reads) at an average coverage of 3x. Bases with Q < 20 were trimmed before read mapping with BWA [31, 32] against the SL2.40 genome sequence of *S. lycopersicum* cv. Heinz with a maximum insert size of 750 bp (50% deviation), reporting at most 30 hits and removing PCR duplicates. SAMTOOLS [31] was used for variant calling without skipping InDels and a minimum gap distance of 5 bp. In addition, GATK [33], was used to call variants for all 60 genotypes in one single analysis.

The JBrowse by Skinner *et al.* [34] was used for the embedding and visualization of the SNP variants. The available gene models (ITAG 2.3) were obtained from the Sol Genomics Network ( http://solgenomics.net/). Subsequently, a script was generated in order to combine the information of SNPs within the RILs. Access to the JBrowse with the information of the sequences can be obtained through: http://www.tomatogenome.net/ril_variants. Furthermore, the program Marker2sequence [35] was used to look for genes between specific genome coordinates based on their annotation.

### TYLCV screening

#### Virus inoculation

*Agrobacterium*-mediated inoculation was performed to infect plants with TYLCV. Plantlets at the 3–4 leaf stage (approximately 21 days after sowing) were inoculated with *A. tumefaciens* LBA4404 bearing a tandem repeat of an infectious TYLCV-IL (Israel isolate) clone. Bacterial growth was performed as previously described by Verlaan *et al.* [36] and bacteria were injected into true leaves using syringes without needle. Plants were grown under greenhouse conditions at 23°C, 60% humidity and 16-h/8-h day/night cycle.

#### Disease test

Disease symptoms were recorded 20, 25, 35 and 45 days post inoculation. Plants were scored for symptom severity according to the scale described by Friedmann *et al.* [37]. A first screening of the RILs was conducted using one plant per line. Thereafter, a second screening followed for the RILs classified as resistant to confirm the phenotype where four plants per resistant line were assessed. TYLCV disease symptoms rating was: 0 = no visible symptoms, inoculated plants show same growth and development as non-inoculated plants; 1 = very slight yellowing and minor curling of leaflet margins on apical leaf; 2 = some yellowing and minor curling of leaflet ends; 3 = a wide range of leaf yellowing, curling and cupping, with some reduction in size, yet plants continue to develop; 4 = very severe plant stunting and yellowing, and pronounced cupping and curling; plants cease to grow (Additional file 1: Figure S2).

#### Metabolic profiling

The RIL population was grown in triplicate under the same greenhouse conditions. Seven weeks after sowing, fully developed leaves were detached and main veins were removed. Samples were frozen in liquid nitrogen and thereafter ground into fine powder.

Untargeted metabolic profiling of leaves was performed with three platforms: 1) Liquid chromatography (LC), using a C18-reversed phase column, coupled to a Quadrupole-time-of-flight (TOF) mass spectrometer (MS) and a photodiode array detector (PDA) to detect semi-polar compounds such as flavonoids, alkaloids, phenylpropanoids, saponins, phenolic acids and polyamines according to De Vos *et al.* [38]. 2) Gas chromatography (GC) coupled to electron impact time of flight (TOF)-MS for detection of primary metabolites according to Lisec *et al.* [39]. 3) Solid phase microextraction (SPME)-GC-MS for the analysis of volatiles according to Tikunov *et al.* [40].

### Metabolomics data processing

Metabolites were quantified and identified according to Tikunov *et al.* [41]. Each dataset was processed using MetAlign ( http://www.metalign.nl) for baseline correction, noise estimation, and ion-wise mass spectral alignment of the corresponding chromatograms. MSClust software was used to extract compounds mass spectra and for data reduction [42].

The putative identification of metabolites was based upon their spectra, retention time, molecular weight and fragmentation patterns. For LC-MS data, compound characteristics were analysed and compared using the Dictionary of Natural Products ( http://dnp.chemnetbase.com) and in-house tomato metabolite databases. GC-MS data were annotated using the NIST Mass Spectral Search Program v2.0 ( http://chemdata.nist.gov/mass-spc/ms-search/) by matching mass spectra extracted to the NIST mass spectra collection and the Golm Metabolome Database ( http://gmd.mpimp-golm.mpg.de/) for mass spectra matching followed by retention index comparison.

### Linkage analysis

Linkage maps were constructed using JoinMap® 4.1 (Kyazma^©^: http://www.kyazma.nl/, [43]) with the specifications by Víquez-Zamora *et al.* [12] using the Haldane’s mapping function. Genetic linkage groups were compared to the physical maps based on the tomato genome version SL2.40 using MapChart 2.2 [44]. The software GenStat 16^th^ edition was used to perform mapping of QTLs for TYLCV resistance and the MapQTL software was used to map metabolite QTLs (mQTLs). The genotypic and phenotypic information is available at: http://www.plantbreeding.wur.nl/Publications/SNP/RILs_genotype-TYLCVphenotype.xlsx. Identified QTLs for TYLCV resistance were named according to their chromosomal position as in Kadirvel *et al*. [45]; *qTy-p3* and *qTy-p11* (*p* as from *S. pimpinellifolium*) for QTL on Chromosomes 3 and 11, respectively. The Marker2sequence application was used to mine regions for candidate genes [35].

Furthermore, the information of the sequences was embedded into JBrowse 1.11.1 [34] to visualize the detected structural variants. The SL2.40 tomato genome assembly and ITAG 2.31 tomato genome annotation was loaded together with the BAM and VCF files of the 60 genotypes.

## Results

### Linkage map and genome-wide visualizations

**Figure 1:**
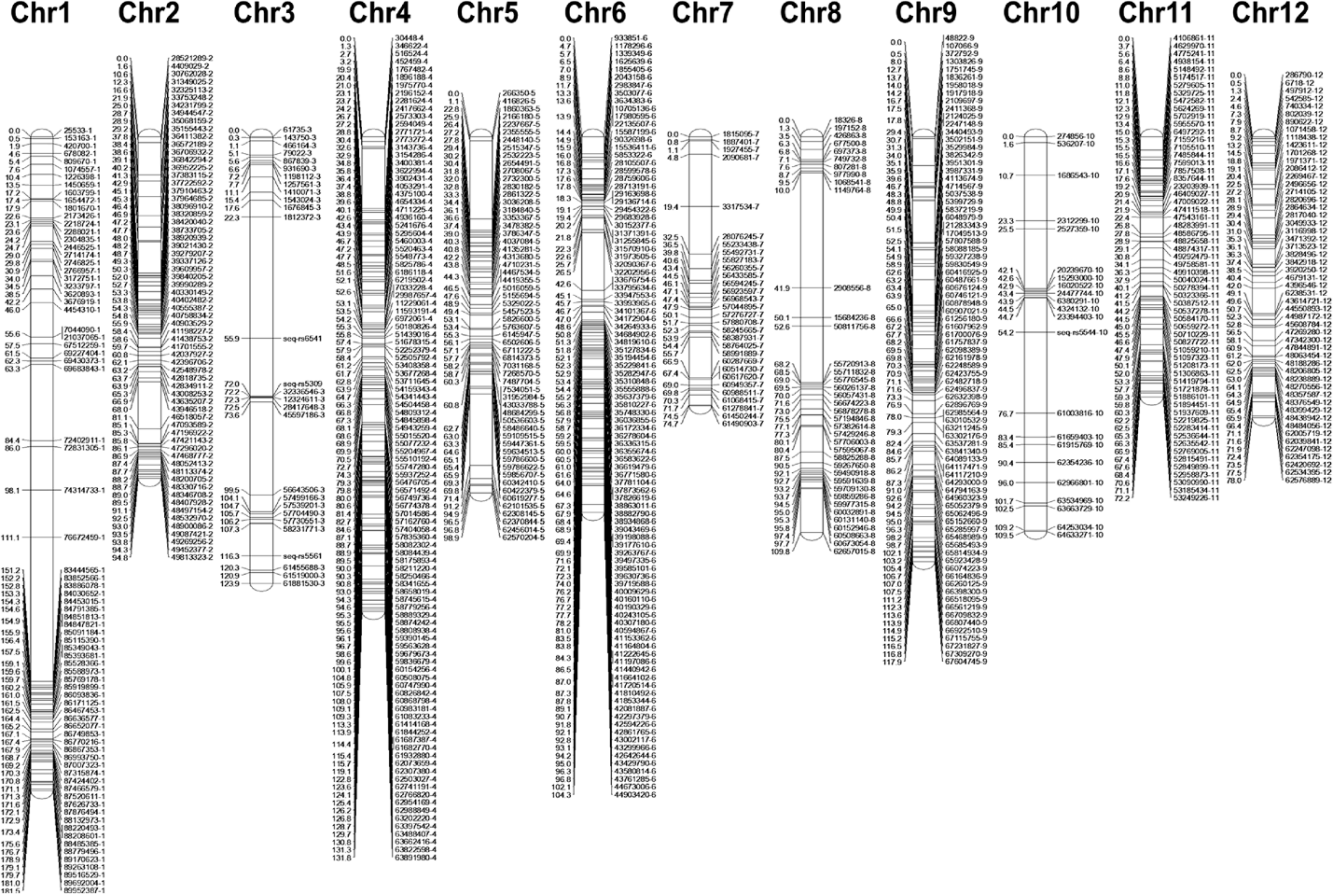
**Linkage map of a RIL population originating from a cross between** ***Solanum lycopersicum*** **cv.** Moneymaker and *Solanum pimpinellifolium* G1.1554. The map shows 715 SNPs representing single recombination positions. Markers are named according to their physical positions.

A custom made SNP Array was assembled from polymorphisms mainly found between two cherry and two round tomatoes [12]. This array was used to genotype a RIL population between *S. lycopersicum* cv. Moneymaker and *S. pimpinellifolium* G1.1554. A total of 1974 polymorphic SNPs were identified between the parents. These SNPs were used to develop a linkage map based on their segregation patterns among the 100 RILs. The resulting map included 715 loci with an average distance of 1.85 cM between loci (Figure 1). The greatest gap was approximately 40 cM on Chromosome 1 and covered the region between 76 and 83 Mb.

**Figure 2:**
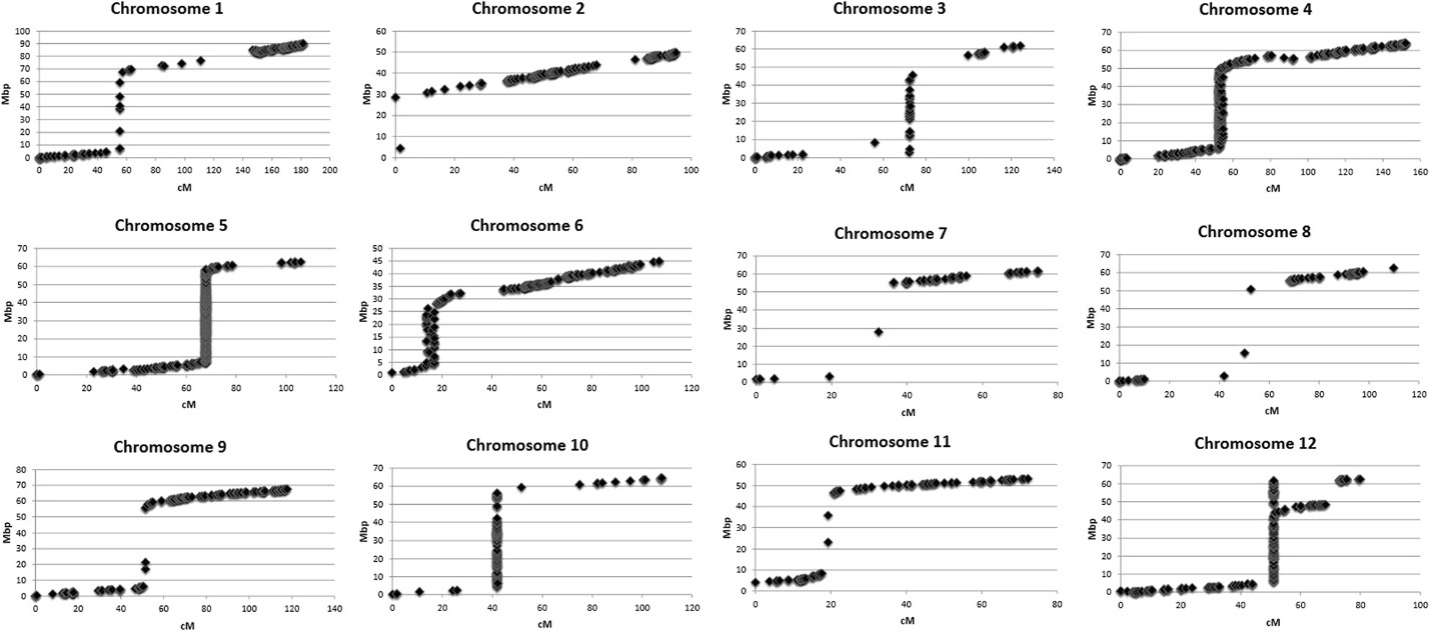
**Scatter plots combining linkage maps (genetical positions in cM) and physical positions (Mb) from the RIL population created from a cross between** ***Solanum lycopersicum*** **cv.** Moneymaker and *Solanum pimpinellifolium* G1.1554.

In order to visualize the recombination patterns along each chromosome, the physical positions of the SNP markers were determined using the published tomato genome [1]. For each SNP and its flanking sequence, a BLAST was performed to the genome sequence version SL2.40. Except for markers on chromosome 12, colinear orders were observed between the genetic and physical maps, as shown in scatter plots per chromosome between the linkage (cM) and physical map (Mb) (Figure 2). These scatter plots further allowed the visualization of cold- and hot-spots of recombination. When a large physical distance corresponds to only a small difference in cM, we can assume cold-spots of recombination. These cold-spots were always the heterochromatin pericentromeric regions and could be as long as 50 to 80 Mb. In contrast, hot-spots of recombination could be present if there is a large cM difference corresponding to small physical distance between markers.

The mosaic pattern of each RIL was calculated and composition of lines varied between 20% and 80% of alleles coming from each parent. In addition, we calculated the SNP allele frequency within the RIL population per marker location along each chromosome. The frequency distribution was mostly 50-50% as expected. However, we found skewness in the distribution of two regions. A preference for *S. pimpinellifolium* alleles was seen near the centromere of Chromosome 2, and a preference for *S. lycopersicum* alleles on Chromosome 9 (Additional file 2: Figure S1).

### *QTLs and*in silico *mapping*

The genotypic file and the linkage map obtained above were then used to map multiple traits. One of the traits screened using our RIL population was TYLCV resistance. Eighty-one RILs were infected with TYLCV. Typical virus symptoms appeared from 30 days after inoculation (dpi); plants were scored according to their symptom development up to 45 dpi and classified as Resistant (R) or Susceptible (S). The susceptible parent ‘Moneymaker’, as expected, displayed severe TYLCV symptoms such as plant stunting and reduced leaf size with upwards curling and yellowing. The resistant parent, *S. pimpinellifolium* G1.1554, remained without symptoms until the end of the experiment. Five out of 81 tested RILs showed no symptoms after virus inoculation (disease score = 0), and four RILs showing very mild symptoms (disease score ≤ 1) were considered resistant. The remaining 72 RILs were classified as susceptible, showing clear TYLCV symptoms including the characteristic leaf curling and yellowing with disease scores ranging from 2 to 4 (Additional file 1: Figure S2).

**Figure 3:**
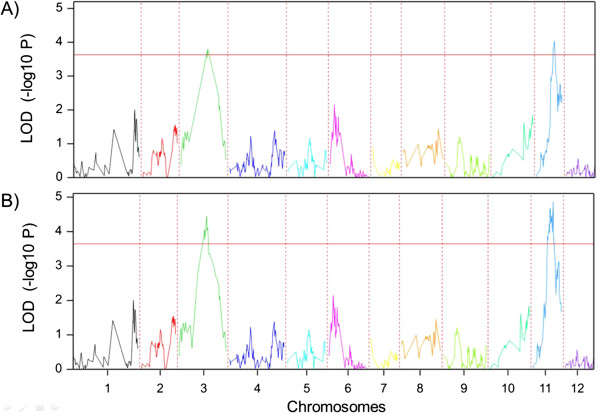
**QTL mapping of** ***qTy-p3*** **and** ***qTy-p11*** **(Chromosome 3 and Chromosome 11) conferring resistance to TYLCV from** ***S. pimpinellifolium*** **G1.1554.** Y-axis represents values according to the interval mapping, horizontal red line delimits threshold of 3.6. **A)** QTL mapping in GenStat only with the SNPs obtained from the SNP array. **B)** QTL mapping after the inclusion of more SNP information obtained from sequences in chromosomes 3 and 11.

In order to identify the genomic regions involved in the resistance, single trait QTL analysis was performed. Two putative QTLs associated with the resistance were identified, one on Chromosome 3, hereafter referred to as *qTy-p3*, and one on Chromosome 11, hereafter referred to as *qTy-p11* (Figure 3A). For *qTy-p3,* 20 markers showed significant association with a LOD value ranging from 3.68 to 3.81, locating the QTL between 4.74 and 45.59 Mb of chromosome 3; the most significant marker for *qTy-p3* was L_45597186-3. For *qTy-p11*, 6 significantly associated markers were identified with a LOD value from 3.79 to 4.04, in a region between 50.82 and 51.20 Mb of chromosome 11. The most significant marker for this QTL was L_51208173-11 (Figure 3; Additional file 3: Figure S3).

Sixty lines from the RIL population were re-sequenced, and the resulting genome sequences were aligned to the published tomato genome, version SL2.40 [1]. The fully resistant lines were included among the 60 sequenced RILs. JBrowse [34] was used to visualize SNP variants within the RILs and allowed us to retrieve the corresponding SNP information of all aligned reads in regions of interest.

We selected 43 additional SNPs to saturate Chromosome 3 resulting in approximately one marker per 0.6 Mb. For Chromosome 11, we included two markers in the region of 7.5-8.3 Mb and 27 in the region between 49-53Mbp. As a result, the Chromosomes 3 and 11 linkage groups were improved, as was the *in silico* mapping for the subset of 60 lines.

The outcome of the QTL analysis with the enriched genotypic data and improved genetic map is depicted in Figure 3B. Using this extended dataset, the analysis confirmed the QTLs *qTy-p3* and *qTy-p11*, The calculated threshold was very similar to the previous calculated threshold (3.64). For *qTy-p3* the LOD values ranged from 3.7 to 4.5, comprising a region with 53 significantly linked markers. The most significantly linked marker position for *qTy-p3* was then refined from 45597186 bp in the first QTL mapping to 46454095 bp and 46520535 bp (both LOD of 4.46) in the improved version. For *qTy-p11* the LOD values for the 26 significantly linked markers (in the improved map) ranged from 3.86 to 4.86, and the most significant marker position was refined from 51208173 bp to 51347236 bp and 51373277 bp (both LOD of 4.86). Together, both QTLs explained almost 28% of the phenotypic effect (13.46 for *qTy-p3* and 14.18 for *qTy-p11*).

A QTL analysis using cofactors (MQM) was performed. When the most significant markers of Chromosome 3 were used as cofactors, the LOD values of *qTy-p11* decreased but were still significant. However when the most significant markers of Chromosome 11 were used as cofactors, the values of *qTy-p3* decreased to non-significant levels. Therefore, the greater impact of *qTy-p11*for the resistance was confirmed. Although all resistant RILs were homozygous for the *S. pimpinellifolium* allele at both QTLs, 14 RILs had disease scores of 2–4 (susceptible). Thus both QTLs with the favourable alleles are necessary for resistance, but their presence did not necessarily result in resistant plants.

### Identification of candidate genes

In order to identify candidate genes for TYLCV resistance, we re-explored the QTL regions using the physical positions of the SNP markers flanking the QTLs. For Chromosome 11, we targeted the region between 50.2 and 51.4 Mb. For *qTy-p11*, a total of 124 predicted genes were identified using Marker2sequence [35] based on the tomato genome sequence (Sol Genomics Network, SGN). Four putative disease-resistance proteins were predicted in the *qTy-p11* region, three of them clustering in the region from position 51347236 to 51373277. Furthermore, approximately 74.9 kb of *qTy-p11* overlaps with the region reported to contain the *Ty-2* resistance allele from *S. habrochaites* accession B6013 [46].

The *qTy-p3* QTL region is physically large, from 2.48 to 47.44 Mb (45 Mb), including the centromeric region. This QTL region harbours more than six hundred annotated genes. In the vicinity of position 46454095 bp (the marker with the highest LOD score) there are genes related to sugars (e.g. high-affinity sugar transporters) and flavonoids (e.g. flavanone 3-hydroxylase-like protein).

### RIL population metabolic profiling

Using the RIL population (not TYLCV infected), we performed untargeted metabolic profiling on leaf material. Primary metabolites were evaluated using GC-TOF-MS. Few differences were observed between parents and individuals of the population showing a similaritiein the primary metabolism. However, the LC-TOF-MS and the SPME-GC-MS platforms uncovered more differences and revealed several QTLs for secondary metabolites and volatiles. More than 200 QTLs were found with putatively identified compounds; an mQTL for sucrose was mapped near *qTy-p11,* and several mQTLs for flavonoid glycosides were present near the region of *qTy-p3* (Additional file 4: Table S1).

Furthermore, since there were TYLCV-susceptible and resistant lines with both QTLs having the homozygous *S. pimpinellifolium* alleles, we performed a T-Test with all metabolic data in order to find metabolites that were significantly different between the two groups of RILs. Five compounds showed significant differences (p-value lower than 0.05) and had higher accumulations in the resistant plants. Three of them were putatively identified as glycosylated forms of kaempferol (LCS146), laricitrin (LCS149) and quercetin (LCS151) having a 4.3, 3.8 and 2.8-fold change, respectively. The other two compounds were acetoxytomatine (C724) and sucrose (C121) with 1.6 and 1.5-fold difference, respectively.

## Discussion

### High-throughput genetic mapping

The custom made SNP array was designed to distinguish different *S. lycopersicum* cultivars, nevertheless a vast amount of polymorphisms were detected between *S. pimpinellifolium* and *S. lycopersicum* cv. Moneymaker making it possible to construct a high density genetic linkage map. In general, positions on the genetic linkage map were consistent with the physical positions on the tomato genome showing the accuracy and robustness of the map and the quality of the tomato sequence.

High and low recombination rates were consistent with the known distribution of euchromatic and heterochromatic regions, as shown by Sim *et al.* [8]. Chromosomes 1, 3, 4, 5 and 10 had large regions without recombination including the centromeres. Centromeric patterns were also observed for chromosomes 6, 7, 8, 9 and 11, but there were some possible distortions that could profit from more markers in the region. Still, the distortions of Chromosome 6 might be influenced by the distinct heterochromatin distribution that follows an alternating pattern [47]. Chromosome 12 also showed a non-recombining centromeric pattern, but this is a clear representation of the likely scaffold misalignment reported previously [12]. Strong clustering of markers on the genetic map but with a clear physical distance between these markers shows a suppression of recombination in these areas (Figure 2).

The allele frequencies showed a preference for the *S. pimpinellifolium* alleles near the centromere on Chromosome 2. This part of the chromosome is linked to rDNA genes. Therefore, there could be a preference for *S. pimpinellifolium* rDNA. A preference was also found for the ‘Moneymaker’ alleles on Chromosome 9 which might be related to deleterious effects of carrying the *S. pimpinellifolium* alleles in this region or to structural DNA differences. Species in the same genus can have DNA configuration differences generating structural changes in the rearrangement of chromosomes after a cross [11]. Differences in local recombination frequencies could be related to the pairing of homologous chromosomes, DNA sequence similarity or divergence, including the presence or absence of genes involved in the recombination process, chromatin conformation or to differences in timing during meiosis [48].

Actual research is enriched by the combination of different software packages. The combination of JBrowse [34], loaded with gene models from the Sol Genomics Network ( http://solgenomics.net/), with previous information of possible genes of interest obtained from Marker2sequence [35] allowed an efficient targeted *in silico* mapping.

### TYLCV resistance mapping and ~ omics platforms combination

The sequenced subset of 60 lines created suitable tools for mapping regions of interest. We enriched regions on Chromosome 3 and Chromosome 11 that were associated with TYLCV resistance, and the *in silico* approach proved to be successful in increasing the power of QTL detection. After the addition of more SNPs coming from the known sequences, we confirmed that *qTy-p3* and *qTy-p11* were not artefacts but had real effects. This allowed us to target the location of the QTL region for *qTy-p11* and it showed the most significant region for *qTy-p3* (Figure 3), even though a large region of Chromosome 3, including the centromere, looks to have an essential impact on the expression of the resistance.

The effect of both QTLs together explained only 28% of the phenotypic effect on the resistance of our RIL population, suggesting additional genetic factors playing a role on the resistance which might have been undetected in our analysis. The accuracy of QTL localization using RILs depends on population size, where a genome-wide coverage of the parents should be present in the mapping population [49]. The fact that both *qTy-p3* and *qTy-p11* were needed for resistance but their presence does not necessarily lead to resistant plants also suggests the possible interaction of extra factors. TYLCV resistance derived from a number of *S. pimpinellifolium* accessions (e.g. LA121, LA373, UPV16991) has been previously suggested to be quantitatively inherited and to show variable gene penetrance [24]. Further genotyping, targeting the regions of low marker coverage, is being assessed in order to detect the presence of one or more additional QTLs, or potential modifier genes. These interactions might be associated with the secondary metabolism of the plants.

A number of TYLCV resistance loci have been reported from different wild *Solanum* species, including *S. chilense*, *S. habrochaites* and *S. peruvianum* [25]. Recently, the *Ty-1* gene from *S. chilense* LA1969 has been cloned and is a representative for a novel class of resistance genes, an RNA-dependent RNA polymerase of the RDRγ class [18, 50]. TYLCV resistance in *S. chilense* accessions LA1932 and LA2779, *S. habrochaites* accession B6013 and TY172, a tomato line derived from different accessions of *S. peruvianum* have been mapped to Chromosomes 3 and 10 (*Ty-4* [25] and *Ty-6* [51]), Chromosome 11 (*Ty-2* [46]) and Chromosome 4 (*ty-5* [16]), respectively.

Several accessions from *S. pimpinellifolium* have been screened and identified to confer resistance to TYLCV [19, 20, 22–25]. However, the genetics of the trait are complex and only one report on mapping resistance originating from *S. pimpinellifolium* (accession ‘Hirsute INRA’) has been reported using RAPD markers [21]. This resistance was mapped to Chromosome 6, close to the *Ty-1* gene. The QTLs identified in the present study represent newly mapped loci conferring resistance derived from *S. pimpinellifolium* G1.1554 and provide a starting point for assessing putative candidate genes in the identified regions. A cluster of disease resistance-like proteins is present near *qTy-p11* (based on the cultivated tomato genome sequence). Furthermore, this region on Chromosome 11 overlaps with 75 kb of the upper part of the mapped region of *Ty-2*, a TYLCV resistance allele derived from *S. habrochaites* accession B6013 [46]. Although *Ty-2* has not yet been cloned, annotated genes in this common region (e.g. elongation factor 1-alpha) might provide further insights for assessing candidate genes for TYLCV resistance derived from these wild tomato species, and/or additional genes involved in the resistance pathway. Plant defense mechanisms are the result of complex gene networks which trigger or mediate the signaling pathways leading to resistance. Besides the reported *Ty*-loci, genes playing a role in these networks have been identified from their differential expression in resistant vs. susceptible genotypes and induced by TYLCV infection, e.g. *Permease I*-like protein and the hexose transporter *LeHT1* [52, 53]. Silencing these genes through Virus-induced gene silencing (VIGS) in a resistant genotype led to the collapse of the resistance, demonstrating the role and importance of these genes in the defense network of the plant.

In general, the presence of compounds such as amino acids and organic acids was very similar between the two species. Differences are more pronounced in the secondary metabolism. Our metabolic data show that the compounds present at higher amounts in the resistant plants are mainly flavonoid glycosides. Flavonoids are phenolic compounds known to be involved in resistance to diverse stress conditions, including plant viruses [54]. For instance quercetin, one of the metabolites detected at higher levels in the resistant lines is a flavonoid known to inhibit HSP70 (Heat-shock protein 70) transcription in animal and plant cells. In *N. benthamiana,* Tomato yellow leaf curl Sardinia virus (TYLCSV) had a delayed infection speed after silencing a member of the HSP70 family, showing that high levels of this protein are required for infection of the virus [55]. Inhibition of HSP70 expression by quercetin resulted in decreased amounts of nuclear TYLCV coat protein in tomato, demonstrating the potential involvement of this flavonoid in the virus resistance pathway [56]. Furthermore, an additional QTL analysis suggests that glycosides of the flavonoid kaempferol co-localise with the TYLCV resistance QTL on Chromosome 3 and that sucrose could be related to the QTL on Chromosome 11 (Additional file 4: Table S1). Kaempferol is known for its antibacterial properties. Besides, we observed the presence of this compound and other flavonoids attached to hexoses in the resistant RILs; transporters of hexoses have been reported to play crucial roles in disease resistance [53, 57]. Some of these compounds likely linked to the resistance also showed an mQTL on chromosome 1 besides the one on chromosome 3, and the mQTL of sucrose also showed significance on chromosome 7. These regions will be further targeted in a fine mapping effort following up this research.

It should be noted that the different concentrations of the compounds observed in resistant vs. susceptible lines were measured prior to TYLCV infection. Sade *et al.* [58] showed that the expression of genes controlling the synthesis of these phenolic compounds is associated with TYLCV resistance. Genes in the flavonoid biosynthesis pathway of a resistant line derived from *S. habrochaites* increased their expression after TYLCV infection leading to the accumulation of flavonoids and contributing to the resistance.

## Conclusion

A RIL population obtained from a cross between *S. lycopersicum* cv. Moneymaker and *S. pimpinellifolium* G1.1554 was successfully genotyped with a custom made SNP array. Furthermore, the re-sequencing of a subset of the RILs allowed the possibility of *in silico* mapping of TYLCV resistance. Two QTLs were related to the resistance, one showing the highest significance on Chromosome 11 close to the region of 51.3 Mb and the other close to 46.5Mbp on Chromosome 3. However, there might be extra loci or genetic factors playing a role that could be unravelled if the population size is increased or when advanced populations are further explored. The resistance towards TYLCV suggests an interaction between flavonoids and hexoses favouring the trait.

We concluded that investments in sequencing can redeem the value of screenings of germplasm due to the fact that both SNPs and sequences can be targeted at the same time. Therefore, screenings can start with a defined number of retrieved SNPs per chromosome, and thereafter, regions of interest can be further targeted. However, data storage, software acquisition and qualified human resources for data analysis and interpretation of combined ~ omics platforms are going to make the difference to get robust analyses.

## Electronic supplementary material

Additional file 1: Figure S2: Disease scores of TYLCV symptom development. Plants were scored according to symptom severity: 0, no visible symptoms; 1, very slight yellowing and minor curling of leaflet margins; 2, yellowing and minor curling of leaflet ends; 3, leaf yellowing, curling and cupping; 4, severe leaf yellowing, curling and cupping, plant stunting (Friedmann *et al.*, [37]). (PPTX 2 MB)

Additional file 2: Figure S1: Probabilities of marker frequencies calculated in GenStat. A skewness in the direction of the chromosome region from *S. pimpinellifolium* G1.1554 is observed for Chromosome 2. A skewness in the direction of the chromosome region from *S. lycopersicum* cv. Moneymaker is observed for Chromosome 9. (PPTX 535 KB)

Additional file 3: Figure S3: Physical map of Chromosome 3 and Chromosome 11 between 49–53 Mb. A) Chromosomes with initial SNPs. B) Chromosomes with incorporated SNPs in green. Black arrows indicate the most significant marker related to TYLCV resistance on each case. Black frames indicate the length covering significant markers for *qTy-p3* and *qTy-p11*. (PNG 2 MB)

Additional file 4: Table S1: QTLs found in non-infected leaves among the population between *S. lycopersicum* cv. Moneymaker and *S. pimpinellifolium* G1.1554. (DOCX 86 KB)
