# Supplementary figures and images for: Mapping in the era of sequencing: high density genotyping and its application for mapping TYLCV resistance in Solanum pimpinellifolium
Source: BMC Genomics. 2014 Dec 20;15(1):1152. doi: 10.1186/1471-2164-15-1152 (PMC4367842; doi:10.1186/1471-2164-15-1152)

## Slide 1
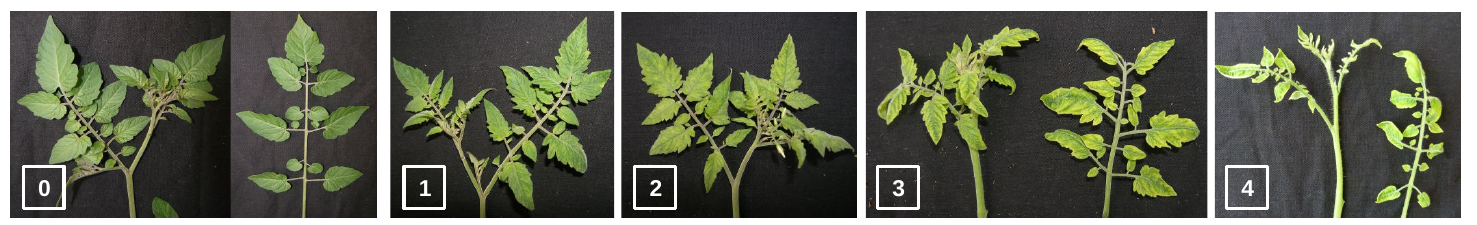

0
1
2
3
4

Supplement: Supplementary file 1 — Additional file 1: Figure S2: Disease scores of TYLCV symptom development. Plants were scored according to symptom severity: 0, no visible symptoms; 1, very slight yellowing and minor curling of leaflet margins; 2, yellowing and minor curling of leaflet ends; 3, leaf yellowing, curling and cupping; 4, severe leaf yellowing, curling and cupping, plant stunting (Friedmann et al., [37]). (PPTX 2 MB) [file 12864_2014_6887_MOESM1_ESM.pptx]

## Slide 1
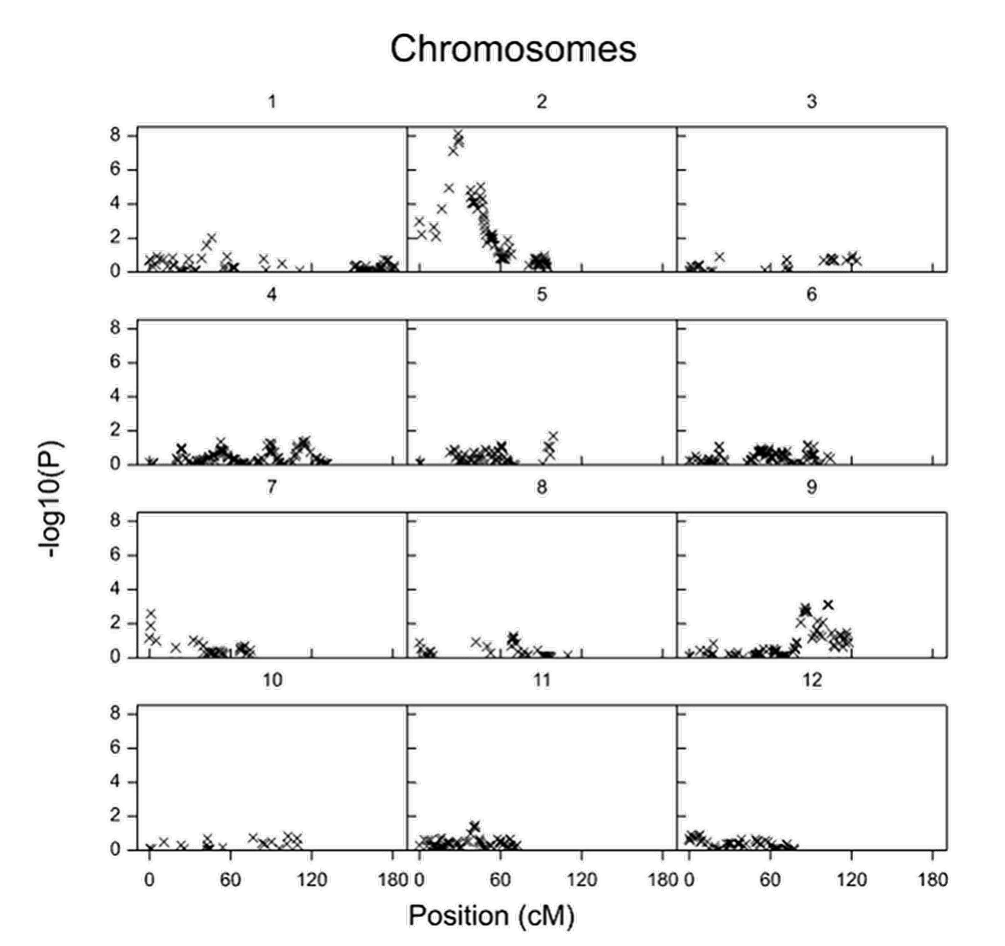

Supplement: Supplementary file 2 — Additional file 2: Figure S1: Probabilities of marker frequencies calculated in GenStat. A skewness in the direction of the chromosome region from S. pimpinellifolium G1.1554 is observed for Chromosome 2. A skewness in the direction of the chromosome region from S. lycopersicum cv. Moneymaker is observed for Chromosome 9. (PPTX 535 KB) [file 12864_2014_6887_MOESM2_ESM.pptx]

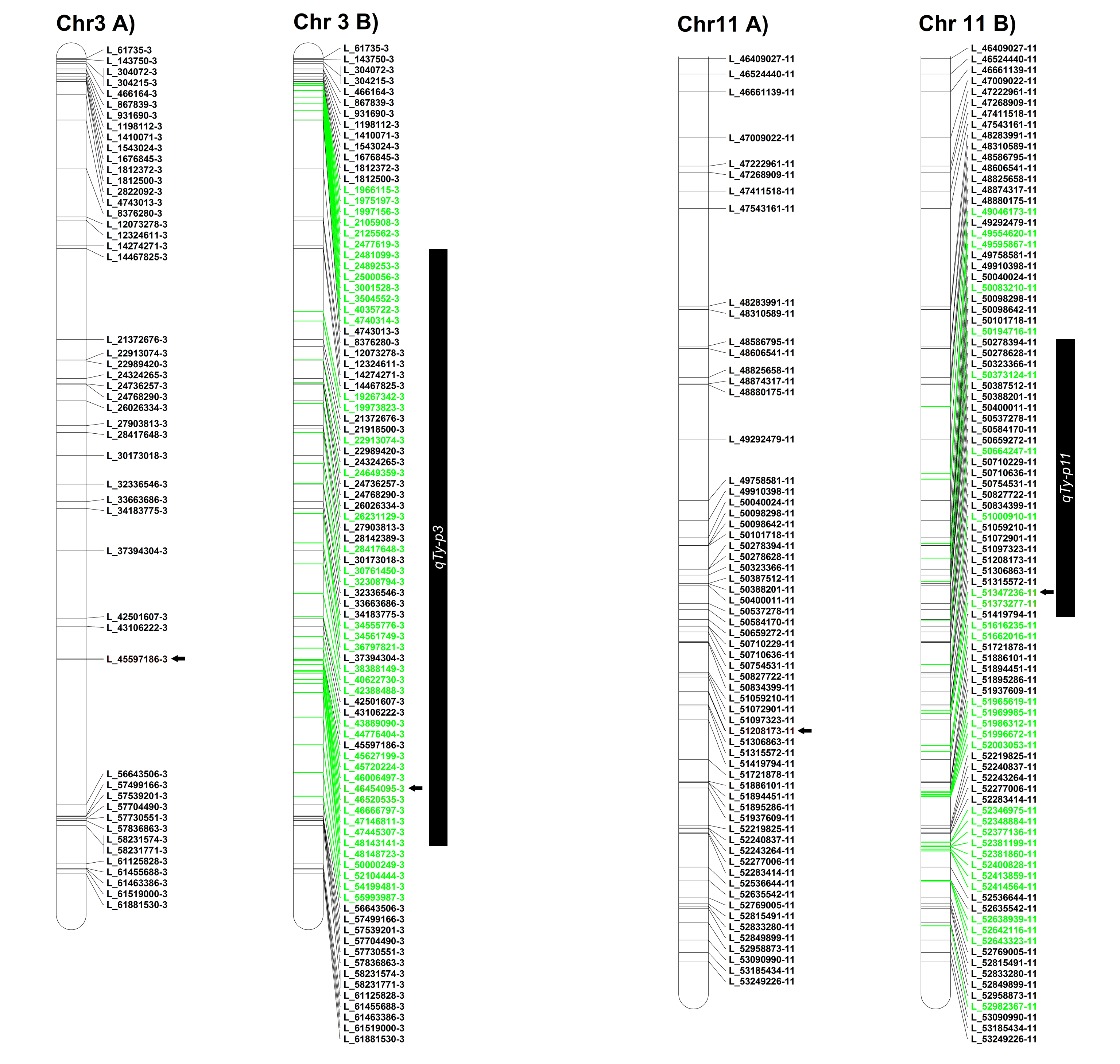

Supplement: Supplementary file 3 — Additional file 3: Figure S3: Physical map of Chromosome 3 and Chromosome 11 between 49–53 Mb. A) Chromosomes with initial SNPs. B) Chromosomes with incorporated SNPs in green. Black arrows indicate the most significant marker related to TYLCV resistance on each case. Black frames indicate the length covering significant markers for qTy-p3 and qTy-p11. (PNG 2 MB) [file 12864_2014_6887_MOESM3_ESM.png]
