# Supplementary material for: Mapping in the era of sequencing: high density genotyping and its application for mapping TYLCV resistance in Solanum pimpinellifolium
Source: BMC Genomics. 2014 Dec 20;15(1):1152. doi: 10.1186/1471-2164-15-1152 (PMC4367842; doi:10.1186/1471-2164-15-1152)
Supplement: Supplementary file 4 — Additional file 4: Table S1: QTLs found in non-infected leaves among the population between S. lycopersicum cv. Moneymaker and S. pimpinellifolium G1.1554. (DOCX 86 KB) [file 12864_2014_6887_MOESM4_ESM.docx]

**Supplemental Table 1.** QTLs found in non-infected leaves among the population between *S. lycopersicum* var. Moneymaker and *S. pimpinellifolium* G1.1554.

| **Putative Compound Identification** | **Identification Level*** | **Specific negative ion, m/z**** | **Compound class** | **Platform** | **Trait Name** | **Chr** | **Position (cM)** | **Marker (closest)** | **LOD** | **% Expl.** |
| --- | --- | --- | --- | --- | --- | --- | --- | --- | --- | --- |
| Delphinidin deoxyhexose-feruloyl-hexose | 5 | 947.2435 | Flavonoid | LC-QTOF-MS | LCS131 | Chr1 | 7.44 | 1074557-1 | 3.01 | 13.30 |
| Cinnamicacid | 5 | 147.0427 | Phenolic Acid | LC-QTOF-MS | LCS2 | Chr1 | 26.69 | 2446525-1 | 3.05 | 13.50 |
| Caryophyllene oxyde | 3 | 161 | Sesquiterpene | GC-SPME-MS | SPME12691 | Chr1 | 36.48 | 3233797-1 | 3.24 | 14.3 |
| Humulene | 2 | 146 | Sesquiterpene | GC-SPME-MS | SPME11646 | Chr1 | 37.48 | 3620893-1 | 4.32 | 18.6 |
| Caryophyllene | 2 | 124 | Sesquiterpene | GC-SPME-MS | SPME11232 | Chr1 | 38.48 | 3620893-1 | 4.57 | 19.5 |
| O-Cymene | 2 | 91 | Terpenoid/Alkylbenzene | GC-SPME-MS | SPME3329 | Chr1 | 38.51 | 3620893-1 | 3.25 | 14.3 |
| Myrcene | 2 | 119 | Terpenoid | GC-SPME-MS | SPME3543 | Chr1 | 40.51 | 3676919-1 | 3.61 | 15.7 |
| Glycoalkaloid | 5 | 1344.6145 | Alkaloid | LC-QTOF-MS | C816 | Chr1 | 51.96 | 7044090-1 | 3.59 | 15.70 |
| Quercetin3-O-rutinoside-7-O-glucoside | 2 | 771.1974 | Flavonoid | LC-QTOF-MS | C365 | Chr1 | 55.58 | 7044090-1 | 3.38 | 14.80 |
| Kaempferol-hexose-hexose-hexose | 5 | 771.1974 | Flavonoid | LC-QTOF-MS | LCS52 | Chr1 | 55.58 | 7044090-1 | 3.49 | 15.30 |
| N296 | 4 | 693.3505 | n.a. | LC-QTOF-MS | C435 | Chr1 | 107.12 | 76672459-1 | 4.15 | 17.90 |
| N740 | 4 | 493.2294 | n.a. | LC-QTOF-MS | LCS171 | Chr1 | 134.15 | 83444565-1 | 9.05 | 34.90 |
| Geraniol | 4 | 51 | Terpenoid | GC-SPME-MS | SPME9044 | Chr1 | 139.15 | 83444565-1 | 5.47 | 22.9 |
| 3-Methyl-2-butenal | 2 | 56 | Leucine/Isoleucine derivative | GC-SPME-MS | SPME774 | Chr1 | 151.15 | 83444565-1 | 4.42 | 18.9 |
| (E)-4-Oxo-2-hexenal | 3 | 57 | Lipid derivative alcohol | GC-SPME-MS | SPME2724 | Chr1 | 152.75 | 83886078-1 | 6.7 | 27.2 |
| (Z)-2-Hexenol | 2 | 100 | Lipid derivative alcohol | GC-SPME-MS | SPME1864 | Chr1 | 152.75 | 83886078-1 | 18.36 | 58.2 |
| 1-Penten-3-one | 1 | 51 | Lipid derivative alcohol | GC-SPME-MS | SPME536 | Chr1 | 152.75 | 83886078-1 | 7.27 | 29.2 |
| 2,2,6-Trimethylcyclohexanone | 2 | 69 | Cyclic molecule | GC-SPME-MS | SPME4755 | Chr1 | 152.75 | 83886078-1 | 6.69 | 27.2 |
| T-2-hexenal | 2 | 84 | Lipid derivative alcohol | GC-SPME-MS | SPME1506 | Chr1 | 152.75 | 83886078-1 | 23.68 | 67.5 |
| (4Z)-Heptenal | 2 | 83 | Lipid derivative alcohol | GC-SPME-MS | SPME2271 | Chr1 | 154.28 | 84453015-1 | 13.11 | 46.3 |
| (E)Hex-3-enol | 1 | 70 | Lipid derivative alcohol | GC-SPME-MS | SPME1557 | Chr1 | 154.28 | 84453015-1 | 14.49 | 49.7 |
| 2-ethylthiophene | 2 | 52 | Heterocyclic compound | GC-SPME-MS | SPME2001 | Chr1 | 154.28 | 84453015-1 | 14.58 | 50 |
| E-2-pentenal | 1 | 85 | Lipid derivative alcohol | GC-SPME-MS | SPME826 | Chr1 | 154.28 | 84453015-1 | 17.24 | 55.9 |
| Pentanal | 1 | 50 | Lipid derivative alcohol | GC-SPME-MS | SPME602 | Chr1 | 154.28 | 84453015-1 | 4.07 | 17.6 |
| (Z)-2-pentenol | 2 | 63 | Lipid derivative alcohol | GC-SPME-MS | SPME944 | Chr1 | 154.34 | 84453015-1 | 7.86 | 31.2 |
| Heptanal | 1 | 71 | Lipid derivative alcohol | GC-SPME-MS | SPME2290 | Chr1 | 154.34 | 84453015-1 | 7.91 | 31.3 |
| Hexa-2,4-dienal | 1 | 61 | Lipid derivative alcohol | GC-SPME-MS | SPME2379 | Chr1 | 154.34 | 84453015-1 | 11.93 | 43.2 |
| Penten-3-ol | 2 | 37 | Lipid derivative alcohol | GC-SPME-MS | SPME486 | Chr1 | 154.34 | 84453015-1 | 9.41 | 36 |
| 3-penten-2-one | 2 | 69 | Lipid derivative alcohol | GC-SPME-MS | SPME744 | Chr1 | 154.60 | 84791385-1 | 3.53 | 15.4 |
| Hexanoic acid, 2-oxo-, methyl ester | 4 | 97 | Carboxilic fatty acid | GC-SPME-MS | SPME4900 | Chr1 | 154.60 | 84791385-1 | 3.87 | 16.8 |
| β-Acoradien-15-ol | 4 | 63 | Sesquiterpene | GC-SPME-MS | SPME10498 | Chr1 | 154.60 | 84791385-1 | 6.56 | 26.8 |
| 2-ethylfuran | 2 | 49 | Lipid derivative alcohol | GC-SPME-MS | SPME621 | Chr1 | 154.86 | 84851813-1 | 12.59 | 45 |
| 3-Hexenoic acid, (E) | 3 | 99 | Fatty acid | GC-SPME-MS | SPME3127 | Chr1 | 154.86 | 84851813-1 | 13.22 | 46.6 |
| 4-methylpentanol | 2 | 41 | Lipid derivative alcohol | GC-SPME-MS | SPME1419 | Chr1 | 154.86 | 84851813-1 | 6.29 | 25.8 |
| Hexanal | 1 | 61 | Lipid derivative alcohol | GC-SPME-MS | SPME1223 | Chr1 | 154.86 | 84851813-1 | 4.16 | 17.9 |
| Pentanol | 1 | 53 | Lipid derivative alcohol | GC-SPME-MS | SPME912 | Chr1 | 154.86 | 84851813-1 | 5.03 | 21.3 |
| Phenylethanal | 1 | 90 | Phenolic | GC-SPME-MS | SPME4932 | Chr1 | 154.86 | 84851813-1 | 5.63 | 23.5 |
| (Z)-2-pentenol | 4 | 67 | Lipid derivative alcohol | GC-SPME-MS | SPME853 | Chr1 | 155.86 | 85091184-1 | 7.96 | 31.5 |
| 2-Heptanol | 2 | 70 | Alcohol | GC-SPME-MS | SPME2235 | Chr1 | 156.44 | 85115390-1 | 3.52 | 15.4 |
| Cis-3-nonen-1-ol | 4 | 45 | Lipid derivative alcohol | GC-SPME-MS | SPME5848 | Chr1 | 157.51 | 85349043-1 | 5.68 | 23.6 |
| Ethyl Acetate | 2 | 62 | Ester | GC-SPME-MS | SPME281 | Chr1 | 157.51 | 85349043-1 | 4.82 | 20.4 |
| Phenol | 1 | 37 | Phenolic | GC-SPME-MS | SPME3091 | Chr1 | 157.51 | 85349043-1 | 5.32 | 22.3 |
| Linalyl oxide | 1 | 72 | Terpenoid | GC-SPME-MS | SPME5527 | Chr1 | 159.12 | 85528366-1 | 5.05 | 21.3 |
| P-mentha-1,5-dien-8-ol | 4 | 68 | Terpenoid | GC-SPME-MS | SPME7758 | Chr1 | 159.12 | 85528366-1 | 4.35 | 18.7 |
| Trans linalool furanoxide | 4 | 111 | Terpenoid | GC-SPME-MS | SPME5785 | Chr1 | 159.12 | 85528366-1 | 4.15 | 17.9 |
| Linalool | 2 | 86 | Terpenoid | GC-SPME-MS | SPME5953 | Chr1 | 160.17 | 85919899-1 | 15.39 | 51.9 |
| α-terpinol | 2 | 62 | Terpenoid | GC-SPME-MS | SPME7947 | Chr1 | 160.17 | 85919899-1 | 8.96 | 34.7 |
| 1-p-Menthen-9-al | 2 | 84 | Terpenoid | GC-SPME-MS | SPME8586 | Chr1 | 160.96 | 86093836-1 | 5.48 | 22.9 |
| Quercetin3-O-glucoside | 1 | 463.0887 | Flavonoid | LC-QTOF-MS | C554 | Chr1 | 161.49 | 86171125-1 | 4.04 | 17.40 |
| Kaempferol-hexose-deoxyhexose,-hexose-coumaroyl | 5 | 901.2403 | Flavonoid | LC-QTOF-MS | LCS146 | Chr1 | 167.14 | 86749853-1 | 4.92 | 20.80 |
| Laricitrin-deoxyhexose-coumaroyl | 5 | 785.1927 | Flavonoid | LC-QTOF-MS | LCS149 | Chr1 | 168.71 | 86993750-1 | 3.64 | 15.90 |
| Quercetin-hexose-deoxyhexose,-hexose, -C10H8O3 (176) | 2 | 947.2434 | Flavonoid | LC-QTOF-MS | C625 | Chr1 | 169.23 | 87007323-1 | 5.13 | 21.60 |
| Quercetin-hexose-deoxyhexose,-hexose,-coumaroyl | 2 | 917.2349 | Flavonoid | LC-QTOF-MS | C643 | Chr1 | 169.23 | 87007323-1 | 6.93 | 28.00 |
| Delphinidin-deoxyhexose-coumaroyl-hexose | 5 | 917.2350 | Flavonoid | LC-QTOF-MS | LCS136 | Chr1 | 169.23 | 87007323-1 | 4.86 | 20.60 |
| Camphene | 4 | 92 | Terpenoid | GC-SPME-MS | SPME8873 | Chr1 | 171.60 | 87626733-1 | 7.95 | 31.4 |
| Isocitricacid | 1 | 191.0191 | organic acid | LC-QTOF-MS | C240 | Chr1 | 178.87 | 89170623-1 | 3.47 | 15.20 |
| N458 | 4 | 623.1622 | n.a. | LC-QTOF-MS | C596 | Chr2 | 20.60 | 33753248-2 | 3.04 | 13.40 |
| Glucose | 3 | 157 | Sugar | GC-TOF-MS | GCTOF6232 | Chr2 | 21.92 | 33753248-2 | 3.05 | 13.4 |
| Glucopyranose | 4 | 204 | Sugar | GC-TOF-MS | GCTOF9109 | Chr2 | 21.92 | 33753248-2 | 3.65 | 15.7 |
| (E)-Geranylacetone | 4 | 109 | Acyclic carotenoids | GC-SPME-MS | SPME11158 | Chr2 | 30.20 | 35155443-2 | 3.5 | 15.3 |
| Eugenol | 1 | 117 | Phenylpropanoid | GC-SPME-MS | SPME10297 | Chr2 | 45.34 | 37964685-2 | 27.22 | 72.5 |
| Eugenol-hexose-pentose | 1 | 457.1724 | Phenylpropanoid glycosilated volatile | LC-QTOF-MS | LCS132 | Chr2 | 46.34 | 38096910-2 | 12.91 | 45.80 |
| Pentadecanal | 2 | 124 | Lipid derivative | GC-SPME-MS | SPME13089 | Chr2 | 48.23 | 39021430-2 | 3.06 | 13.5 |
| Tridecanal | 4 | 79 | Lipid derivative | GC-SPME-MS | SPME13102 | Chr2 | 48.23 | 39021430-2 | 3.03 | 13.4 |
| Quercetin-dihexose-deoxyhexose | 5 | 771.1979 | Flavonoid | LC-QTOF-MS | LCS79 | Chr2 | 77.96 | 46518057-2 | 3.68 | 16.00 |
| Kaempferol-hexose-deoxyhexose,-hexose-coumaroyl | 2 | 901.2407 | Flavonoid | LC-QTOF-MS | C728 | Chr2 | 89.51 | 48407928-2 | 3.47 | 15.20 |
| Methylbutenol | 2 | 68 | Leucine/Isoleucine derivative | GC-SPME-MS | SPME254 | Chr2 | 91.12 | 48497154-2 | 3.56 | 15.5 |
| Acetoxy-tomatine+FA | 1 | 1136.5490 | Alkaloid | LC-QTOF-MS | C724 | Chr2 | 94.81 | 49813323-2 | 3.63 | 15.80 |
| 4-Oxoisophorone | 2 | 152 | Cyclic ketone | GC-SPME-MS | SPME6959 | Chr3 | 77.05 | 46454095-3 | 4.79 | 20.3 |
| Methylbutenol | 2 | 68 | Leucine/Isoleucine derivative | GC-SPME-MS | SPME254 | Chr3 | 77.05 | 46454095-3 | 3.16 | 13.9 |
| N238 | 4 | 431.1921 | n.a. | LC-QTOF-MS | C416 | Chr3 | 80.31 | 47146811-3 | 4.15 | 17.90 |
| 1-Nonanol | 4 | 98 | Lipid derivative alcohol | GC-SPME-MS | SPME7260 | Chr3 | 93.81 | 54199481-3 | 3.06 | 13.5 |
| Geraniol | 4 | 51 | Terpenoid | GC-SPME-MS | SPME9044 | Chr3 | 97.33 | 54199481-3 | 3.51 | 15.3 |
| Isopentanol | 1 | 54 | Leucine/Isoleucine derivative | GC-SPME-MS | SPME702 | Chr3 | 101.33 | 55993987-3 | 5.09 | 21.5 |
| Laricitrin-hexose,hexose | 3 | 665.1724 | Flavonoid | LC-QTOF-MS | LCS88 | Chr3 | 111.25 | 57499166-3 | 3.16 | 13.90 |
| Quercetin-deoxyhexose-feruloyl | 5 | 785.1929 | Flavonoid | LC-QTOF-MS | LCS151 | Chr3 | 111.25 | 57499166-3 | 4.07 | 17.60 |
| N338 | 4 | 793.1805 | n.a. | LC-QTOF-MS | LCS102 | Chr3 | 113.37 | 57730551-3 | 4.78 | 20.30 |
| Quercetin-3-O-glucoside | 1 | 463.0887 | Flavonoid | LC-QTOF-MS | C554 | Chr3 | 114.43 | 58231574-3 | 3.02 | 13.40 |
| Kaempferol-3-O-rutinoside | 1 | 593.1501 | Flavonoid | LC-QTOF-MS | C585 | Chr3 | 114.43 | 58231574-3 | 3.61 | 15.80 |
| Quercetin-hexose-deoxyhexose,-hexose, -C10H8O3 (176) | 2 | 947.2434 | Flavonoid | LC-QTOF-MS | C625 | Chr3 | 114.43 | 58231574-3 | 3.02 | 13.40 |
| Quercetin-hexose-deoxyhexose, -C12H12O5(236) | 2 | 845.2148 | Flavonoid | LC-QTOF-MS | C773 | Chr3 | 114.43 | 58231574-3 | 3.51 | 15.40 |
| N458 | 4 | 623.1622 | n.a. | LC-QTOF-MS | C596 | Chr3 | 114.43 | 58231574-3 | 3.79 | 16.50 |
| Quercetin-hexose,-hexose (3,7-O) | 5 | 625.1405 | Flavonoid | LC-QTOF-MS | C362 | Chr3 | 114.43 | 58231574-3 | 4.06 | 17.50 |
| N429 | 5 | 773.1933 | n.a. | LC-QTOF-MS | C466 | Chr3 | 114.43 | 58231574-3 | 3.60 | 15.70 |
| Kaempferol-3-O-glucoside | 1 | 447.0937 | Flavonoid | LC-QTOF-MS | C601 | Chr3 | 115.43 | 58231574-3 | 4.86 | 20.60 |
| Quercetin-hexose-deoxyhexose,-pentose | 2 | 741.1871 | Flavonoid | LC-QTOF-MS | C473 | Chr3 | 115.43 | 58231574-3 | 6.83 | 27.70 |
| Kaempferol-hexose | 5 | 447.0937 | Flavonoid | LC-QTOF-MS | LCS125 | Chr3 | 115.43 | 58231574-3 | 4.67 | 19.90 |
| Kaempferol-hexose-deoxyhexose,-pentose | 2 | 725.1921 | Flavonoid | LC-QTOF-MS | LCS101 | Chr3 | 116.43 | 58231574-3 | 6.64 | 27.10 |
| Laricitrin-deoxyhexose-coumaroyl | 5 | 785.1927 | Flavonoid | LC-QTOF-MS | LCS149 | Chr3 | 116.43 | 58231574-3 | 4.89 | 20.70 |
| Kaempferol3-O-rutinoside-7-O-glucoside | 1 | 755.2031 | Flavonoid | LC-QTOF-MS | LCS71 | Chr3 | 117.43 | 58231574-3 | 6.01 | 24.80 |
| Kaempferol3-O-rutinoside | 5 | 593.1516 | Flavonoid | LC-QTOF-MS | C406 | Chr3 | 117.43 | 58231574-3 | 6.00 | 24.80 |
| Kaempferol -hexose-deoxyhexose,-hexose-coumaroyl | 5 | 901.2403 | Flavonoid | LC-QTOF-MS | LCS146 | Chr3 | 117.43 | 58231574-3 | 8.92 | 34.50 |
| Heptahydroxyflavone,-trimethylether | 3 | 375.0712 | Flavonoid | LC-QTOF-MS | LCS53 | Chr4 | 92.78 | 58658019-4 | 3.36 | 14.80 |
| P-mentha-1,5-dien-8-ol | 4 | 94 | Terpenoid | GC-SPME-MS | SPME7486 | Chr4 | 99.56 | 59836679-4 | 3.23 | 14.2 |
| 1,3,8-p-Menthatriene | 4 | 78 | Terpenoid | GC-SPME-MS | SPME5859 | Chr4 | 99.62 | 59836679-4 | 3.94 | 17 |
| Quercetin-hexose-deoxyhexose,-pentose | 2 | 741.1871 | Flavonoid | LC-QTOF-MS | C473 | Chr4 | 125.10 | 62954169-4 | 3.14 | 13.80 |
| Methyl salicylate | 1 | 104 | Phenylpropanoid | GC-SPME-MS | SPME8127 | Chr4 | 125.44 | 62954169-4 | 3.75 | 16.3 |
| Hexanoic acid, 2-oxo-, methyl ester | 4 | 97 | Carboxilic fatty acid | GC-SPME-MS | SPME4900 | Chr5 | 38.98 | 3786347-5 | 3.27 | 14.4 |
| Threitol | 5 | 103 | Sugar alcohol | GC-TOF-MS | GCTOF2469 | Chr5 | 38.98 | 3786347-5 | 3.18 | 13.9 |
| N-Acetylglutamic acid | 1 | 174 | Amino acid | GC-TOF-MS | GCTOF2777 | Chr5 | 38.98 | 3786347-5 | 3.39 | 14.7 |
| Pentanal | 1 | 50 | Lipid derivative alcohol | GC-SPME-MS | SPME602 | Chr5 | 90.42 | 62101535-5 | 3.34 | 14.7 |
| L-Glutamic acid | 1 | 246 | Amino acid | GC-TOF-MS | GCTOF3032 | Chr5 | 91.18 | 62101535-5 | 3.26 | 14.2 |
| Methylheptenone | 4 | 77 | Organic compound | GC-SPME-MS | SPME3411 | Chr5 | 96.75 | 62456014-5 | 3.86 | 16.8 |
| Benzophenone | 2 | 181 | Phenolic ketone | GC-SPME-MS | SPME12882 | Chr6 | 16.83 | 28105507-6 | 3.08 | 13.6 |
| Quercetin-hexose-deoxyhexose,-hexose,-coumaroyl | 2 | 917.2349 | Flavonoid | LC-QTOF-MS | C643 | Chr6 | 46.97 | 34172904-6 | 3.07 | 13.60 |
| Isopentanol | 1 | 54 | Leucine/Isoleucine derivative | GC-SPME-MS | SPME702 | Chr6 | 52.86 | 35282947-6 | 3.41 | 14.9 |
| Pentanol | 1 | 53 | Lipid derivative alcohol | GC-SPME-MS | SPME912 | Chr6 | 52.86 | 35282947-6 | 3.39 | 14.9 |
| 4-methylpentanol | 2 | 41 | Lipid derivative alcohol | GC-SPME-MS | SPME1419 | Chr6 | 69.43 | 39198088-6 | 3.94 | 17 |
| Laricitrin-hexose,hexose | 3 | 665.1724 | Flavonoid | LC-QTOF-MS | LCS88 | Chr6 | 89.11 | 42081887-6 | 3.59 | 15.70 |
| (E)Hex-3-enol | 1 | 70 | Lipid derivative alcohol | GC-SPME-MS | SPME1557 | Chr7 | 21.43 | 3317534-7 | 4.4 | 18.9 |
| Sucrose | 1 | 341.1074 | Sugar | LC-QTOF-MS | C121 | Chr7 | 70.35 | 61068415-7 | 5.82 | 24.20 |
| Fenchene | 4 | 52 | Terpenoid | GC-SPME-MS | SPME6919 | Chr8 | 2.34 | 197152-8 | 3.89 | 16.9 |
| 1,3,8-p-Menthatriene | 4 | 78 | Terpenoid | GC-SPME-MS | SPME5859 | Chr8 | 3.34 | 426863-8 | 8.93 | 34.5 |
| (E)-Ocimene | 2 | 66 | Terpenoid | GC-SPME-MS | SPME4844 | Chr8 | 3.53 | 426863-8 | 15.37 | 51.8 |
| 2-Carene | 2 | 66 | Terpenoid | GC-SPME-MS | SPME3962 | Chr8 | 3.53 | 426863-8 | 22.67 | 65.9 |
| Camphene | 2 | 107 | Terpenoid | GC-SPME-MS | SPME2895 | Chr8 | 3.53 | 426863-8 | 6.58 | 26.8 |
| Limonene | 1 | 65 | Terpenoid | GC-SPME-MS | SPME4484 | Chr8 | 3.53 | 426863-8 | 11.01 | 40.7 |
| P-Cymen-9-ol | 2 | 132 | Terpenoid | GC-SPME-MS | SPME7806 | Chr8 | 3.53 | 426863-8 | 6.7 | 27.2 |
| P-Cymol | 2 | 66 | Terpenoid | GC-SPME-MS | SPME4397 | Chr8 | 3.53 | 426863-8 | 19.42 | 60.2 |
| Pinene | 2 | 74 | Terpenoid | GC-SPME-MS | SPME2621 | Chr8 | 3.53 | 426863-8 | 5.15 | 21.7 |
| Pinene | 4 | 136 | Terpenoid | GC-SPME-MS | SPME6601 | Chr8 | 3.53 | 426863-8 | 13.24 | 46.7 |
| P-mentha-1,5-dien-8-ol | 4 | 94 | Terpenoid | GC-SPME-MS | SPME7486 | Chr8 | 3.53 | 426863-8 | 9.72 | 37 |
| Verbenone | 4 | 108 | Terpenoid | GC-SPME-MS | SPME9327 | Chr8 | 3.53 | 426863-8 | 6.78 | 27.5 |
| α-Phellandrene | 2 | 107 | Terpenoid | GC-SPME-MS | SPME4012 | Chr8 | 3.53 | 426863-8 | 25.74 | 70.5 |
| α-Terpinene | 2 | 119 | Terpenoid | GC-SPME-MS | SPME4204 | Chr8 | 3.53 | 426863-8 | 20.65 | 62.5 |
| β-Phellandrene | 2 | 123 | Terpenoid | GC-SPME-MS | SPME4643 | Chr8 | 3.53 | 426863-8 | 27.11 | 72.4 |
| O-Cymene | 2 | 50 | Terpenoid/Alkylbenzene | GC-SPME-MS | SPME3280 | Chr8 | 4.53 | 426863-8 | 5.98 | 24.7 |
| O-Cymene | 2 | 91 | Terpenoid/Alkylbenzene | GC-SPME-MS | SPME3329 | Chr8 | 4.53 | 426863-8 | 3.77 | 16.4 |
| P-mentha-1,5-dien-8-ol | 4 | 51 | Terpenoid | GC-SPME-MS | SPME7353 | Chr8 | 4.53 | 426863-8 | 6.88 | 27.9 |
| L-Glutamine | 5 | 145.0609 | Amino acid | LC-QTOF-MS | C88 | Chr8 | 70.02 | 56057431-8 | 3.69 | 16.10 |
| LycoperosideHorHydroxytomatineIV +FA | 5 | 1094.5402 | Alkaloid | LC-QTOF-MS | C567 | Chr8 | 72.64 | 56878278-8 | 3.23 | 14.20 |
| Citricacid | 1 | 191.0200 | organic acid | LC-QTOF-MS | C291 | Chr8 | 75.46 | 57194846-8 | 3.16 | 13.90 |
| 3-Caffeoylquinicacid (Chlorogenicacid) | 5 | 353.0876 | phenolic acid | LC-QTOF-MS | C395 | Chr8 | 75.46 | 57194846-8 | 3.04 | 13.40 |
| LycoperosideHorHydroxytomatineI | 5 | 1048.5354 | Alkaloid | LC-QTOF-MS | C765 | Chr8 | 83.37 | 57595067-8 | 3.58 | 15.60 |
| Dehydrotomatine (S)I | 5 | 1076.5258 | Alkaloid | LC-QTOF-MS | C652 | Chr8 | 88.46 | 58825288-8 | 3.01 | 13.30 |
| Benzylalcohol-hexose-pentose | 2 | 401.1456 | Phenolic glycosilated volatile | LC-QTOF-MS | C380 | Chr8 | 94.51 | 59977315-8 | 3.22 | 14.20 |
| Protocatechuicacid | 5 | 153.0204 | phenolic acid | LC-QTOF-MS | C311 | Chr8 | 94.51 | 59977315-8 | 3.65 | 15.90 |
| α-tomatin | 1 | 1078.5415 | Alkaloid | LC-QTOF-MS | C734 | Chr8 | 97.69 | 60673054-8 | 3.32 | 14.60 |
| Tomatidinedihexosedipentose +FA | 1 | 1048.5322 | Alkaloid | LC-QTOF-MS | C749 | Chr8 | 97.69 | 60673054-8 | 3.28 | 14.40 |
| Glycoalkaloid | 5 | 1344.6145 | Alkaloid | LC-QTOF-MS | C816 | Chr8 | 97.69 | 60673054-8 | 3.32 | 14.60 |
| β-Damascenone | 2 | 190 | Cyclic carotenoids | GC-SPME-MS | SPME10714 | Chr9 | 6.52 | 1303826-9 | 4.2 | 18.1 |
| (Z)-2-Hexenol | 2 | 100 | Lipid derivative alcohol | GC-SPME-MS | SPME1864 | Chr9 | 8.02 | 1303826-9 | 4.34 | 18.6 |
| 2-ethylthiophene | 2 | 52 | Heterocyclic compound | GC-SPME-MS | SPME2001 | Chr9 | 8.02 | 1303826-9 | 3.08 | 13.6 |
| E-2-pentenal | 1 | 85 | Lipid derivative alcohol | GC-SPME-MS | SPME826 | Chr9 | 8.02 | 1303826-9 | 3.69 | 16.1 |
| L-Aspartic acid | 1 | 100 | Amino acid | GC-TOF-MS | GCTOF2612 | Chr9 | 38.88 | 4113674-9 | 4.21 | 17.9 |
| Phenylethanol | 1 | 37 | Aromatic alcohol | GC-SPME-MS | SPME6326 | Chr9 | 52.47 | 57807588-9 | 6.15 | 25.3 |
| Quercetin-hexose,-hexose (3,7-O) | 5 | 625.1405 | Flavonoid | LC-QTOF-MS | C362 | Chr9 | 63.94 | 60746121-9 | 4.29 | 18.40 |
| Kaempferol-hexose-deoxyhexose,-hexose-coumaroyl | 2 | 901.2407 | Flavonoid | LC-QTOF-MS | C728 | Chr9 | 66.64 | 61256180-9 | 3.16 | 13.90 |
| N152 | 4 | 443.1924 | n.a. | LC-QTOF-MS | LCS41 | Chr9 | 67.16 | 61607962-9 | 6.97 | 28.20 |
| (E)-Geranylacetone | 4 | 109 | Acyclic carotenoids | GC-SPME-MS | SPME11158 | Chr9 | 69.21 | 62098389-9 | 3.05 | 13.5 |
| Laricitrin-deoxyhexose-coumaroyl | 5 | 785.1927 | Flavonoid | LC-QTOF-MS | LCS149 | Chr9 | 70.33 | 62248589-9 | 3.16 | 13.90 |
| Kaempferol-3-O-rutinoside-7-O-glucoside | 1 | 755.2031 | Flavonoid | LC-QTOF-MS | LCS71 | Chr9 | 70.85 | 62423755-9 | 9.64 | 36.70 |
| Quercetin3-O-rutinoside-7-O-glucoside | 2 | 771.1974 | Flavonoid | LC-QTOF-MS | C365 | Chr9 | 70.85 | 62423755-9 | 12.99 | 46.00 |
| Quercetin-dihexose-deoxyhexose-pentose | 3 | 903.2413 | Flavonoid | LC-QTOF-MS | LCS54 | Chr9 | 70.85 | 62423755-9 | 12.84 | 45.60 |
| Quercetin-dihexose-deoxyhexose-pentose | 5 | 903.2408 | Flavonoid | LC-QTOF-MS | C353 | Chr9 | 70.85 | 62423755-9 | 9.99 | 37.80 |
| Kaempferol3-O-rutinoside | 5 | 593.1516 | Flavonoid | LC-QTOF-MS | C406 | Chr9 | 70.85 | 62423755-9 | 10.15 | 38.20 |
| Kaempferol-hexose-hexose-hexose | 5 | 771.1974 | Flavonoid | LC-QTOF-MS | LCS52 | Chr9 | 70.85 | 62423755-9 | 12.04 | 43.50 |
| Isorhamnetin-hexose-hexose (3-O) | 3 | 639.1605 | Flavonoid | LC-QTOF-MS | LCS111 | Chr9 | 76.87 | 62896769-9 | 3.06 | 13.50 |
| Isorhamnetin-hexose,-hexose (3,7-O) | 5 | 639.1574 | Flavonoid | LC-QTOF-MS | LCS107 | Chr9 | 76.87 | 62896769-9 | 3.53 | 15.40 |
| Hexanol-pentose-hexose | 3 | 395.1934 | Lipid glycosilated volatile | LC-QTOF-MS | C572 | Chr9 | 104.21 | 65923428-9 | 3.86 | 16.80 |
| Glycoalkaloid | 5 | 1344.6145 | Alkaloid | LC-QTOF-MS | C816 | Chr9 | 104.21 | 65923428-9 | 3.16 | 13.90 |
| Hexanoic acid, 2-oxo-, methyl ester | 4 | 97 | Carboxilic fatty acid | GC-SPME-MS | SPME4900 | Chr9 | 105.40 | 66074223-9 | 3.45 | 15.1 |
| β-Acoradien-15-ol | 4 | 63 | Sesquiterpenes | GC-SPME-MS | SPME10498 | Chr9 | 105.40 | 66074223-9 | 3.13 | 13.8 |
| N427 | 5 | 773.1922 | n.a. | LC-QTOF-MS | C480 | Chr9 | 106.40 | 66164836-9 | 4.63 | 19.80 |
| 5-Caffeoylquinicacid | 1 | 353.0875 | phenolic acid | LC-QTOF-MS | C337 | Chr10 | 1.00 | 536207-10 | 3.03 | 13.40 |
| 4-Caffeoylquinicacid | 5 | 353.0883 | Acid | LC-QTOF-MS | C361 | Chr10 | 1.00 | 536207-10 | 8.44 | 33.00 |
| Citricacid | 1 | 191.0200 | organic acid | LC-QTOF-MS | C291 | Chr10 | 3.62 | 536207-10 | 3.72 | 16.20 |
| N143 | 5 | 402.9155 | n.a. | LC-QTOF-MS | C29 | Chr10 | 18.65 | 2312299-10 | 3.09 | 13.70 |
| 2-methylbutanol | 1 | 45 | Leucine/Isoleucine derivative | GC-SPME-MS | SPME726 | Chr10 | 23.33 | 2312299-10 | 3.71 | 16.2 |
| Butanol | 2 | 55 | Alcohol | GC-SPME-MS | SPME429 | Chr10 | 23.33 | 2312299-10 | 3.47 | 15.2 |
| Methylbutenol | 2 | 68 | Leucine/Isoleucine derivative | GC-SPME-MS | SPME254 | Chr10 | 23.33 | 2312299-10 | 3.66 | 16 |
| Caffeicacid | 5 | 179.0362 | organic acid | LC-QTOF-MS | C295 | Chr10 | 25.33 | 2527359-10 | 6.42 | 26.30 |
| Kaempferol-hexose-deoxyhexose,-hexose-coumaroyl | 2 | 901.2407 | Flavonoid | LC-QTOF-MS | C728 | Chr10 | 25.52 | 2527359-10 | 4.22 | 18.20 |
| N71 | 4 | 337.0771 | n.a. | LC-QTOF-MS | LCS20 | Chr10 | 42.90 | 16020522-10 | 3.46 | 15.20 |
| β-Damascenone | 2 | 190 | Cyclic carotenoids | GC-SPME-MS | SPME10714 | Chr10 | 44.48 | 4324132-10 | 8.36 | 32.8 |
| 3-methyl-2-butenol | 2 | 68 | Leucine/Isoleucine derivative | GC-SPME-MS | SPME1019 | Chr10 | 47.74 | 23394403-10 | 3.52 | 15.4 |
| Limonene | 1 | 65 | Terpenoid | GC-SPME-MS | SPME4484 | Chr10 | 49.74 | seq-rs5544-10 | 3.84 | 16.7 |
| Coumaroylquinicacid | 1 | 337.0940 | phenolic acid | LC-QTOF-MS | C414 | Chr10 | 62.21 | 59477572-10 | 4.51 | 19.30 |
| Coumaroylquinicacid | 5 | 337.0936 | phenolic acid | LC-QTOF-MS | C462 | Chr10 | 63.21 | 59477572-10 | 4.56 | 19.40 |
| 4-Oxoisophorone | 2 | 152 | Cyclic ketone | GC-SPME-MS | SPME6959 | Chr10 | 95.36 | 62966801-10 | 3.18 | 14 |
| 2-ethylhexanol | 2 | 81 | Lipid derivative alcohol | GC-SPME-MS | SPME4243 | Chr11 | 0.00 | 4106861-11 | 9.81 | 37.2 |
| 2-methylbutanol | 1 | 45 | Leucine/Isoleucine derivative | GC-SPME-MS | SPME726 | Chr11 | 0.00 | 4106861-11 | 10.82 | 40.2 |
| 3-methyl-2-butenol | 2 | 68 | Leucine/Isoleucine derivative | GC-SPME-MS | SPME1019 | Chr11 | 0.00 | 4106861-11 | 3.2 | 14.1 |
| Butanol | 2 | 55 | Alcohol | GC-SPME-MS | SPME429 | Chr11 | 0.00 | 4106861-11 | 11.66 | 42.5 |
| Geranial | 2 | 137 | Terpenoid | GC-SPME-MS | SPME9389 | Chr11 | 0.00 | 4106861-11 | 3.3 | 14.5 |
| Heptanol | 2 | 68 | Lipid derivative alcohol | GC-SPME-MS | SPME2968 | Chr11 | 0.00 | 4106861-11 | 4.78 | 20.3 |
| Hexanol | 1 | 70 | Lipid derivative alcohol | GC-SPME-MS | SPME1892 | Chr11 | 0.00 | 4106861-11 | 6.12 | 25.2 |
| Isopentanol | 1 | 54 | Leucine/Isoleucine derivative | GC-SPME-MS | SPME702 | Chr11 | 0.00 | 4106861-11 | 7.54 | 30.1 |
| Methylbutenol | 2 | 68 | Leucine/Isoleucine derivative | GC-SPME-MS | SPME254 | Chr11 | 0.00 | 4106861-11 | 3.48 | 15.2 |
| Pentanol | 1 | 53 | Lipid derivative alcohol | GC-SPME-MS | SPME912 | Chr11 | 0.00 | 4106861-11 | 7.3 | 29.3 |
| Benzylalcohol-hexose-pentose | 2 | 401.1456 | Phenolic glycosilated volatile | LC-QTOF-MS | C380 | Chr11 | 0.00 | 4106861-11 | 5.60 | 23.30 |
| Xylose | 1 | 103 | Sugar | GC-TOF-MS | GCTOF3183 | Chr11 | 3.71 | 4629970-11 | 3.16 | 13.8 |
| 2-Heptanol | 2 | 70 | Alcohol | GC-SPME-MS | SPME2235 | Chr11 | 8.86 | 5174517-11 | 4.23 | 18.2 |
| Caryophyllene | 2 | 124 | Sesquiterpenes | GC-SPME-MS | SPME11232 | Chr11 | 11.07 | 5279605-11 | 3.58 | 15.6 |
| Caryophyllene oxyde | 3 | 161 | Sesquiterpenes | GC-SPME-MS | SPME12691 | Chr11 | 11.07 | 5279605-11 | 3.78 | 16.4 |
| Humulene | 2 | 146 | Sesquiterpenes | GC-SPME-MS | SPME11646 | Chr11 | 11.07 | 5279605-11 | 3.11 | 13.7 |
| Benzaldehyde | 1 | 63 | Phenolic/Aromatic aldehyde | GC-SPME-MS | SPME2995 | Chr11 | 11.86 | 5329725-11 | 4.02 | 17.4 |
| β-Ionone | 2 | 145 | Cyclic carotenoids | GC-SPME-MS | SPME11902 | Chr11 | 18.82 | 23203939-11 | 3.13 | 13.8 |
| _O-Feruloylquinicacid | 3 | 367.1035 | phenolic acid | LC-QTOF-MS | C449 | Chr11 | 21.02 | 47009022-11 | 3.90 | 16.90 |
| 3-O-Feruloylquinicacid | 5 | 367.1040 | Acid | LC-QTOF-MS | C491 | Chr11 | 21.02 | 47009022-11 | 3.42 | 15.00 |
| Methyl salicylate | 1 | 104 | Phenylpropanoid | GC-SPME-MS | SPME8127 | Chr11 | 46.45 | 50710636-11 | 3.74 | 16.3 |
| N50 | 4 | 609.1888 | n.a. | LC-QTOF-MS | LCS17 | Chr11 | 56.66 | 51347236-11 | 3.75 | 16.30 |
| Sucrose | 1 | 341.1074 | Sugar | LC-QTOF-MS | C121 | Chr11 | 73.08 | 52635542-11 | 4.26 | 18.30 |
| Protocatechuicacid | 5 | 153.0204 | phenolic acid | LC-QTOF-MS | C311 | Chr11 | 73.08 | 52635542-11 | 3.68 | 16.00 |
| 1-Nonanol | 4 | 98 | Lipid derivative alcohol | GC-SPME-MS | SPME7260 | Chr12 | 48.99 | 6238531-12 | 3.31 | 14.5 |
| Octanol | 2 | 71 | Lipid-derived | GC-SPME-MS | SPME5262 | Chr12 | 49.12 | 6238531-12 | 4.2 | 18.1 |
| Quercetin3-O-rutinoside | 1 | 609.1450 | Flavonoid | LC-QTOF-MS | C512 | Chr12 | 49.12 | 6238531-12 | 4.04 | 17.50 |
| Kaempferol3-O-rutinoside | 1 | 593.1501 | Flavonoid | LC-QTOF-MS | C585 | Chr12 | 51.70 | 44987172-12 | 3.42 | 15.00 |
| Kaempferol-3-O-glucoside | 1 | 447.0937 | Flavonoid | LC-QTOF-MS | C601 | Chr12 | 51.70 | 44987172-12 | 4.28 | 18.40 |
| N429 | 5 | 773.1933 | n.a. | LC-QTOF-MS | C466 | Chr12 | 51.70 | 44987172-12 | 3.37 | 14.80 |
| Kaempferol-hexose | 5 | 447.0937 | Flavonoid | LC-QTOF-MS | LCS125 | Chr12 | 51.70 | 44987172-12 | 4.29 | 18.40 |
| Petunidin-deoxyHexose-coumaroyl-hexose +H2O | 5 | 949.2614 | Flavonoid | LC-QTOF-MS | LCS89 | Chr12 | 52.32 | 44987172-12 | 3.21 | 14.10 |
| Guaiacol | 1 | 37 | Phenylpropanoid | GC-SPME-MS | SPME5805 | Chr12 | 74.45 | 62420692-12 | 5.65 | 23.5 |

*Annotation level: 1=Identified compounds. 2=Putatively annotated compounds (e.g. without chemical reference standards, based upon physicochemical properties and/or spectral similarity with public/commercial spectral libraries). 3=Putatively characterized compound classes (e.g. based upon characteristic physicochemical properties of a chemical class of compounds, or by spectral similarity to known compounds of a chemical class). 4. Unknown compounds—although unidentified or unclassified these metabolites can still be differentiated and quantified based upon spectral data. 5. Unknown compounds—similar mass to a putatively characterized compound.

**Compounds analysed using LC-QTOF-MS platform represented by measured accurate masses of corresponding negatively charged parent molecule ions or their formic acid adducts (denoted by +FA). Volatile compounds and primary metabolites measured by SPME-GC-MS and GC-TOF-MS, respectively, represented by selected nominal negative mass ion fragments picked automatically by MSClust software.
